# Novel *Candidatus* Rickettsia Species Detected in Nostril Tick from Human, Gabon, 2014

**DOI:** 10.3201/eid2102.141048

**Published:** 2015-02

**Authors:** Rogelio Lopez-Velez, Ana M. Palomar, José A. Oteo, Francesca F. Norman, José A. Pérez-Molina, Aránzazu Portillo

**Affiliations:** University Hospital Ramón y Cajal, Madrid, Spain (R. Lopez-Velez, F.F. Norman, J.A. Pérez-Molina);; Hospital San Pedro–Center of Biomedical Research of La Rioja, Logroño, Spain (A.M. Palomar, J.A. Oteo, A. Portillo)

**Keywords:** Amblyomma sp., Rickettsia, Candidatus Rickettsia davousti, nostril tick, tick, Gabon, Africa, bacteria, Africa, novel, tickborne diseases, Candidatus Rickettsia species

## Abstract

We report the identification of a nymphal nostril tick (*Amblyomma* sp.) from a national park visitor in Gabon and subsequent molecular detection and characterization of tickborne bacteria. Our findings provide evidence of a potentially new *Rickettsia* sp. circulating in Africa and indicate that tick bites may pose a risk to persons visiting parks in the region.

Ticks are hematophagous arthropods that parasitize different species of vertebrates, and they serve as intermediate hosts for infectious pathogens that can have serious implications for humans. Because of climate change and socioeconomic factors, tickborne diseases have increased in the past 3 decades, and these arthropods are second only to mosquitoes as vectors of human infectious diseases (*1*,*2*). Many ixodid tick species are found in Africa, and tickborne diseases in travelers returning from that continent have been reported worldwide (*3*). Among the travel-associated cases of African tick-bite fever, most occur in persons returning from travel to southern Africa with fever and systemic illness (*4*).

## The Study

A 21-year-old female field worker from Spain visited Lopé National Park in Gabon (Africa) for 13 days during January–February 2014 to observe chimpanzees and gorillas. Four days before returning to Spain, she noticed a foreign body (black spot) inside her left nostril but had no signs or symptoms of illness. After returning home, the woman sought care at the Tropical Medicine Centre at University Hospital Ramón y Cajal in Madrid, Spain, where a tick attached to the anteroinferior part of the left nasal septum (the Kiesselbach area) was extracted with forceps during rhinofibroscopy. The tick was sent to the Center of Rickettsiosis and Arthropod-Borne Diseases at Hospital San Pedro–Center of Biomedical Research of La Rioja in Logroño, Spain, for identification and molecular detection of tickborne bacteria. The tick was photographed (Figure) and identified, by morphologic features, as an *Amblyomma* sp. nymph, according to taxonomic keys (*5*).

**Figure:**
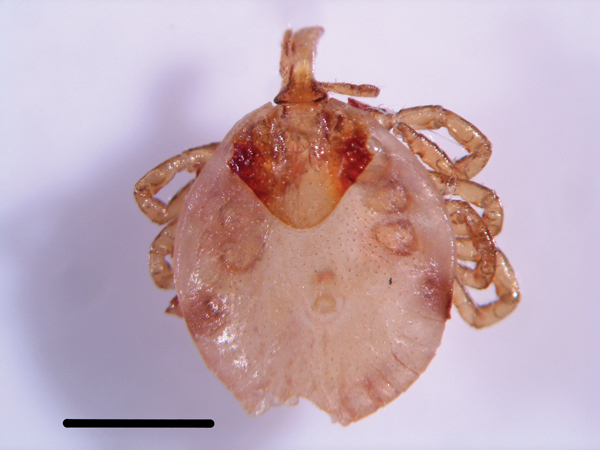
*Amblyomma* sp. nymphal tick removed from the nostril of a woman who visited Lopé National Park in Gabon (Africa), 2014. Scale bar represents 1 mm.

Immature stages of *Amblyomma* ticks cannot be identified to the species level on the basis of morphologic features without allowing the nymph to molt. Thus, we conducted genetic analysis to identify the tick. To extract genomic DNA, we incubated the tick with ammonium hydroxide (1 mL of 25% ammonia and 19 mL of sterile water) for 20 min at 100°C and for another 20 min at 90°C. The DNA was used as template in PCR assays targeting the tick mitochondrial 16S rRNA (*6*), mitochondrial 12S rRNA (*7*), and nuclear 5.8S-28S rRNA intergenic transcribed spacer 2 (ITS2) (*8*). As a positive control, we used DNA extract from a tick of known identity (*Haemaphysalis punctata*) that was collected in La Rioja, Spain.

Subsequent detection and molecular characterization of tickborne bacteria (*Rickettsia* spp., *Anaplasma phagocytophilum*, and *Borrelia* spp.) were performed. To screen for the presence of rickettsiae, we used PCR assays targeting 2 fragments of the *ompB* gene (511 bp and 811 bp, respectively) (*9*,*10*). Four additional genetic markers were used to classify the isolated rickettsia to the species level: fragments of *ompA* (532 bp) (*11*,*12*), 16S rRNA (1,500 bp) (*13*), *sca4* (623 bp) (*14*), and *gltA* (1,019 bp) (*15*). These markers were amplified in accordance with the taxonomic scheme for classifying rickettsiae at the genus and species levels (Technical Appendix, reference *16*).

To screen for the presence of *A. phagocytophilum* and *Borrelia* spp., we performed PCR targeting the partial *msp2* gene (334 bp) and *Borrelia* genus–specific 16S rRNA (1,350 bp) gene (Technical Appendix, references *17,18*). Each PCR included a positive control: *Rickettsia slovaca* strain S14ab DNA (obtained from Vero cells that had been inoculated in our facility with homogenate of an *R. slovaca–*infected *Dermacentor marginatus* tick from La Rioja Province); *A. phagocytophilum* strain Webster DNA (provided by D. Raoult [Unité de Recherche sur les Maladies Infectieuses et Tropicales Emergentes, Marseille, France] and J.S. Dumler [Johns Hopkins Hospital, Baltimore, MD, USA]); or *Borrelia burgdorferi* sensu stricto DNA (provided by V. Fingerle [German National Reference Centre for *Borrelia*, Oberschleissheim, Germany]). DNA-free water was included as a negative control in each set of reactions.

We used BLAST (http://www.ncbi.nlm.nih.gov/blast/Blast.cgi) to compare sequences generated by each pair of primers with sequences in GenBank. The 16S rRNA sequence showed highest identity (91% [368/405 bp]) with the mitochondrion *Amblyomma variegatum* 16S rRNA gene (GenBank accession no. L34312). The 12S rRNA and ITS2 sequences reached only 89% and 91% identity, respectively, with those of *A. variegatum* and were closest (94.3% [298/316 bp] and 99% identity [809/817 bp], respectively) to those for an *Amblyomma* sp. nymph (GenBank accession nos. KC538944 and KC538941). Of interest, the 12S rRNA and ITS2 sequences also corresponded to those of a nostril tick removed from a researcher who had been visiting a national park in Uganda (Technical Appendix, reference *19*); the 16S rRNA sequence for the tick from this researcher was not in GenBank. The levels of sequence similarities that we found did not enable species determination of the tick in this study. Two previous reports about nostril ticks in humans who have visited Africa are available (Technical Appendix, references *20,21*).

For the strain in this study, single bands of the expected sizes for the 2 *ompB* rickettsial fragment genes analyzed were detected. A BLAST search revealed that these 2 sequences were genetically most similar (97.2% and 98.3% identity) to the *ompB* gene of *Rickettsia japonica* and *Rickettsia heilongjiangensis*, respectively (Table). The nucleotide sequence of *ompA* was closest (99.8% identity) to that of the *ompA* of *Rickettsia* sp. strain Davousti, and showed maximum identity (97.2%) with *R. heilogjiangensis* as validated species. When compared with sequences of validly published *Rickettsia* spp. available in GenBank, the 16S rRNA and *sca4* gene sequences showed the highest identity with *R. japonica* (99.4%–99.6% and 98.5%, respectively). The *gltA* sequence shared 99.1% identity with *R. japonica* and *R. heilogjiangensis* (Table). These results are in accordance with the genetic criteria for identifying the rickettsia as *Candidatus* Rickettsia sp. (Technical Appendix, reference *16*).

**Table:** Maximum identities of rickettsial sequences obtained from an *Amblyomma* sp. tick from Gabon with validated *Rickettsia* spp. published in GenBank*

Gene sequence, GenBank accession no.	% identity with *Rickettsia* spp. (basepairs)†		
	*R. japonica*, GenBank accession no. AP011533	*R. heilongjiangensis*, GenBank accession no. CP002912	*Rickettsia* sp. Davousti, GenBank accession nos. DQ402516–402517
*ompB*, KJ619633	97.2 (442/455)	96.9 (441/455)	NA
*ompB*, KJ619632	96.9 (746/770)	98.3 (742/755)	NA
*ompA*, KJ619631	96.1 (472/491)	97.2 (477/491)	99.8 (490/491)
16S rRNA, KJ619629	99.4–99.6 (1369–1373/1378)	99.3–99.6 (1368–1372/1378)	NA
*sca4*, KJ619634	98.5 (509/517)	98.3 (508/517)	NA
*gltA*, KJ619635	99.1 (903/911)	99.1 (903/911)	99.9 (691/692)

*The tick was removed in 2014 from the nostril of a woman who returned home to Spain after visiting Lopé National Park in Gabon. NA, not available. †Percentages of identity with sequences of *Rickettsia* sp. Davousti have been also included when available due to the high level of similarity with our sequences.

Although reports about the circulation of *Rickettsia* spp. in Gabon are scarce, *Rickettsia* sp. strain Davousti was detected in *Amblyomma tholloni* ticks from African elephants in that country (Technical Appendix, reference *22*). In addition, the *gltA* sequence obtained in our study was 99.9% identical to the *gltA* sequence of *Rickettsia* sp. strain Davousti (Table). These findings suggest that both strains could belong to the same *Rickettsia* sp. No other sequences, apart from those for *ompA* and *gltA*, of *Rickettsia* sp. strain Davousti have been deposited in GenBank; however, on the basis of findings in the previous report (Technical Appendix, reference *22*), we propose the name *Candidatus.* R. davousti for the strain in this study.

In addition, we did not obtain amplicons for the *msp2* gene of *A. phagocytophilum* or 16S rRNA gene specific for *Borrelia* genus. For each set of PCR primers, no bands were detected on agarose gels for negative control samples.

The partial tick mitochondrial 16S rDNA, 12S rDNA, and ITS2 sequences have been deposited in GenBank under accession nos. KJ619630, KJ619636, and KJ619637, respectively. The partial *ompB* (2 fragment genes), *ompA*, 16S rRNA, *sca4*, and *gltA* sequences of the novel tick-derived rickettsia in this study have been deposited in GenBank under accession numbers KJ619632, KJ619633, KJ619631, KJ619629, KJ619634, and KJ619635.

## Conclusions

We report the detection of a potentially novel *Rickettsia* sp. from an *Amblyomma* sp. nymphal tick that was removed from the nostril of a field researcher when she returned to Spain after visiting Gabon’s Lopé National Park; we propose the name *Ca*. R. davousti for this *Rickettsia* sp. strain. Our findings provide further evidence of the presence of circulating *Rickettsia* sp. in Africa and indicate that tick bites may be a threat to persons visiting national parks in Africa. Further studies are needed to determine the prevalence of *Ca*. R. davousti and to establish whether this bacterium is pathogenic for humans.

Technical Appendix Additional references for this article.

## References

[1] Sonenshine DE, Lane RS, Nicholson WL. Ticks (Ixodida). In: Mullen G, Durden L, editors. Medical and veterinary entomology. New York: Academic Press; 2002. p 517–58.

[2] Godfrey ER, Randolph SE. Economic downturn results in tick-borne disease upsurge. Parasit Vectors. 2011;4:35. 10.1186/1756-3305-4-35 PMC3063212 21406086

[3] Portillo A, Oteo JA. Rickettsiosis as threat for the traveller. In: Rodriguez-Morales A, editor. Current topics in tropical medicine [cited 2014 Jun 18]. InTech, 2012. http://www.intechopen.com/books/current-topics-in-tropical-medicine/rickettsiosis-as-threat-for-the-traveller

[4] Mendelson M, Han PV, Vincent P, von Sonnenburg F, Cramer JP, Loutan L, GeoSentinel Surveillance Network. Regional variation in travel-related illness acquired in Africa, March 1997–May 2011. Emerg Infect Dis. 2014;20:532–41. 10.3201/eid2004.131128 24655358 PMC3966389

[5] Walker AR, Bouattour A, Camicas JL, Estrada-Peña A, Horak IG, Latif AA, Ticks of domestic animals in Africa: a guide to identification of species. In: Edinburgh (UK): Bioscience Reports; 2003.

[6] Black WC, Piesman J. Phylogeny of hard- and soft-tick taxa (Acari: Ixodida) based on mitochondrial 16S rDNA sequences. Proc Natl Acad Sci U S A. 1994;91:10034–8. 10.1073/pnas.91.21.10034 7937832 PMC44952

[7] Beati L, Keirans JE. Analysis of the systematic relationships among ticks of the genera *Rhipicephalus* and *Boophilus* (Acari: Ixodidae) based on mitochondrial 12S ribosomal DNA gene sequences and morphological characters. J Parasitol. 2001;87:32–48. 10.1645/0022-3395(2001)087[0032:AOTSRA]2.0.CO;2 11227901

[8] Zahler M, Gothe R, Rinder H. Genetic evidence against a morphologically suggestive conspecificity of *Dermacentor reticulatus* and *Dermacentor marginatus* (Acari: Ixodidae). Int J Parasitol. 1995;25:1413–9. 10.1016/0020-7519(95)00081-X 8719952

[9] Choi YJ, Jang WJ, Kim JY, Ryu JS, Lee SH, Park KH, Spotted fever group and typhus group rickettsioses in humans, South Korea. Emerg Infect Dis. 2005;11:237–44. 10.3201/eid1102.040603 15752441 PMC3320442

[10] Roux V, Raoult D. Phylogenetic analysis of members of the genus *Rickettsia* using the gene encoding the outer-membrane protein rOmpB (*ompB*). Int J Syst Evol Microbiol. 2000;50:1449–55. 10.1099/00207713-50-4-1449 10939649

[11] Regnery RL, Spruill CL, Plikaytis BD. Genotypic identification of rickettsiae and estimation of intraspecies sequence divergence for portions of two rickettsial genes. J Bacteriol. 1991;173:1576–89 . 1671856 10.1128/jb.173.5.1576-1589.1991 PMC207306

[12] Roux V, Fournier PE, Raoult D. Differentiation of spotted fever group rickettsiae by sequencing and analysis of restriction fragment length polymorphism of PCR-amplified DNA of the gene encoding the protein rOmpA. J Clin Microbiol. 1996;34:2058–65 . 8862558 10.1128/jcm.34.9.2058-2065.1996 PMC229190

[13] Weisburg WG, Barns SM, Pelletier DA, Lane DJ. 16S ribosomal DNA amplification for phylogenetic study. J Bacteriol. 1991;173:697–703 . 1987160 10.1128/jb.173.2.697-703.1991 PMC207061

[14] Sekeyova Z, Roux V, Raoult D. Phylogeny of *Rickettsia* spp. inferred by comparing sequences of ‘gene D’, which encodes an intracytoplasmic protein. Int J Syst Evol Microbiol. 2001;51:1353–60 . 11491333 10.1099/00207713-51-4-1353

[15] Jado I, Oteo JA, Aldámiz M, Gil H, Escudero R, Ibarra V, *Rickettsia monacensis* and human disease, Spain. Emerg Infect Dis. 2007;13:1405–7. 10.3201/eid1309.060186 18252123 PMC2857266

