# Supplementary material for: Novel Candidatus Rickettsia Species Detected in Nostril Tick from Human, Gabon, 2014
Source: Emerg Infect Dis. 2015 Feb;21(2):325–7. doi: 10.3201/eid2102.141048 (PMC4313649; doi:10.3201/eid2102.141048)
Supplement: Technical Appendix — Additional references for this article. [file 14-1048-Techapp-s1.pdf]

DOI: http://dx.doi.org/10.3201/eid2102.141048

# Novel *Candidatus* Rickettsia Species Detected in Human-Derived Nostril Tick, Gabon, 2014

## Technical Appendix

### References

16. Raoult D, Fournier PE, Eremeeva M, Graves S, Kelly PJ, Oteo JA, et al. Naming of rickettsiae and rickettsial diseases. Ann N Y Acad Sci. 2005;1063:1–12. PubMed http://dx.doi.org/10.1196/annals.1355.002

17. Zeidner NS, Burkot TR, Massung R. Transmission of the agent of human granulocytic ehrlichiosis by *Ixodes spinipalpis* ticks: evidence of an enzootic cycle of dual infection with *Borrelia burgdorferi* in northern Colorado. J Infect Dis. 2000;182:616–9. PubMed http://dx.doi.org/10.1086/315715

18. Raoult D, Ndihokubwayo JB, Tissot-Dupont H, Roux V, Faugere B, Abegbinni R, et al. Outbreak of epidemic typhus associated with trench fever in Burundi. Lancet. 1998;352:353–8. PubMed http://dx.doi.org/10.1016/S0140-6736(97)12433-3

19. Hamer SA, Bernard AB, Donovan RM, Hartel JA, Wrangham RW, Otali E, et al. Coincident tick infestations in the nostrils of wild chimpanzees and a human in Uganda. Am J Trop Med Hyg. 2013;89:924–7. PubMed http://dx.doi.org/10.4269/ajtmh.13-0081

20. Walton GA. A tick infesting the nostrils of man. Nature. 1960;188:1131–2. PubMed http://dx.doi.org/10.1038/1881131a0

21. Aronsen GP, Robbins RG. An instance of tick feeding to repletion inside a human nostril. Bulletin of the Peabody Museum of Natural History. 2008;49:245–8. http://dx.doi.org/10.3374/0079-032X-49.2.245

22. Matsumoto K, Parola P, Rolain JM, Jeffery K, Raoult D. Detection of "*Rickettsia* sp. strain Uilenbergi" and "*Rickettsia* sp. strain Davousti" in *Amblyomma tholloni* ticks from elephants in Africa. BMC Microbiol. 2007;7:74. PubMed http://dx.doi.org/10.1186/1471-2180-7-74
